# Robot-Assisted Dental Implant Surgery Following Guided Bone Regeneration: A Clinical Case Report

**DOI:** 10.1155/crid/2569238

**Published:** 2025-09-29

**Authors:** You Jiajia, Maria Costanza Soldini, Ramón Pons, Cristina Valles

**Affiliations:** ^1^Department of Implantology, Private Practice, Dr. Ya's Dental Clinic, Suzhou, China; ^2^Department of Periodontology, School of Dentistry, International University of Catalonia (UIC), Barcelona, Spain

## Abstract

**Background:**

Accurate placement of dental osseointegrated implants is critical for long-term success, with factors significantly influencing stability and integration with surrounding tissues (i.e., depth, angle, and positioning within the bone). Nowadays, dental surgeries assisted by implant robots are gaining popularity. This occurs because dental robots provide precise and accurate minimally invasive surgery.

**Methods:**

A 46-year-old woman visited a private dental clinic with missing teeth in positions 4.5 and 4.6. Upon clinical and radiographic examination, severe ridge atrophy was observed. To address this, horizontal and vertical guided bone regeneration (GBR) was performed using a customized titanium occlusive membrane. After 6 months, DICOM files of the patients were imported into the robot implant planning software (RemebotDent, Remebot, Beijing Rui Yi Bo Technology DICOM Ltd., China) for the placement of two dental implants (CLC CONIC, CLC Scientific, Vicenza, Italy).

**Results:**

After the GBR procedure, vertical bone gain was on average 2 mm, whereas horizontal bone gain was 5.28 and 5.34 mm in the middle position of 4.5 and 4.6. The robot-assisted surgery revealed high accuracy, since the implants in positions 4.5 and 4.6 showed a coronal global deviation of 0.42 and 0.57 mm and angular deviation of 0.62° and 0.69°, respectively.

**Conclusions:**

This robotic surgical system can offer high accuracy and reliability with the preoperative plan. The potential for future collaboration between robots and surgeons may significantly alter the landscape of implant dentistry, and further studies are needed to confirm the accuracy of robotic implant placement.

**Summary**

• This robotic surgical system ensures precise and reliable preoperative planning, potentially transforming implant dentistry through future collaboration between robots and surgeons.

## 1. Introduction

Implant therapy is a reliable choice for restoring missing teeth in patients who are either fully or partially edentulous [1, 2]. Prosthetically guided implant placement is considered the gold standard in dental implant treatment [3]. In this context, comprehensive pretreatment planning is essential to achieve precise three-dimensional positioning of the implant within the alveolar bone, considering surrounding anatomical structures and future prosthetic restorations [3].

In the last two decades, precise implant placement was improved by the development of static computer-assisted implant surgery (s-CAIS) and, later, by dynamic computer-assisted implant surgery (d-CAIS) [3, 4]. In this sense, s-CAIS relies on preoperative planning and static guides, which may not address intraoperative anatomical variations or unexpected alterations. In contrast, d-CAIS offers real-time feedback and adjustments during surgery, but the surgery still needs to be performed manually, and it requires surgeons to overcome a significant learning curve [5]. These technologies offer several advantages for clinicians but also come with notable limitations, such as high costs and limited accessibility, the need for extensive training for surgeons and staff, potential technical failures (hardware malfunctions, software glitches, or calibration errors), and an overdependence on technology.

In 2021, the National Medical Products Administration in China approved an autonomous robot-assisted surgery system named “Remebot” for dental implant procedures. This system falls under the category of semiactive and task-autonomy robotic systems. It automates the process of implant osteotomy and placement using image-guided, robot-assisted technologies [6].

Oral implant robots can be categorized into two control modes: semiautomatic and automatic types [7]. Semiactive robots perform the drilling, but the implant placement is guided by the surgeon. If the drill deviates from the planned position, the robot system actively restrains it to the intended direction using real-time force feedback [8]. In contrast, the automatic robotic system autonomously drills and places the implant [9].

The use of robotic computer-assisted implant surgery (r-CAIS) has shown more accuracy compared to previous meta-analyses of the s-CAIS and d-CAIS technologies [10, 11]. Additionally, the superior accuracy of robotic surgery was demonstrated in a recent randomized clinical trial that evaluated 31 implants in partially edentulous patients. The study reported an average angle deviation of 2.81 ± 1.13° and 3D deviations of 0.53 ± 0.23 and 0.53 ± 0.24 mm at the level of the implant shoulder and apex, respectively [11]. The results in fully edentulous patients using zygomatic implants are similar, and the robotic surgery exhibited a global coronal deviation of 0.78 ± 0.34 mm, a global apical deviation of 0.80 ± 0.25 mm, and an angular deviation of 1.33° ± 0.71 [12].

This case is aimed at showing the precision achieved when utilizing automatic robotic technology for placing dental implants in the regenerated bone of a partially edentulous patient.

## 2. Materials and Methods

### 2.1. Preoperative Analysis

The present case report was written according to the CARE guidelines (https://www.care-statement.org/). In this sense, data were obtained and used according to ethical requirements; the patient gave her informed consent for the case publication.

**Figure 1:**
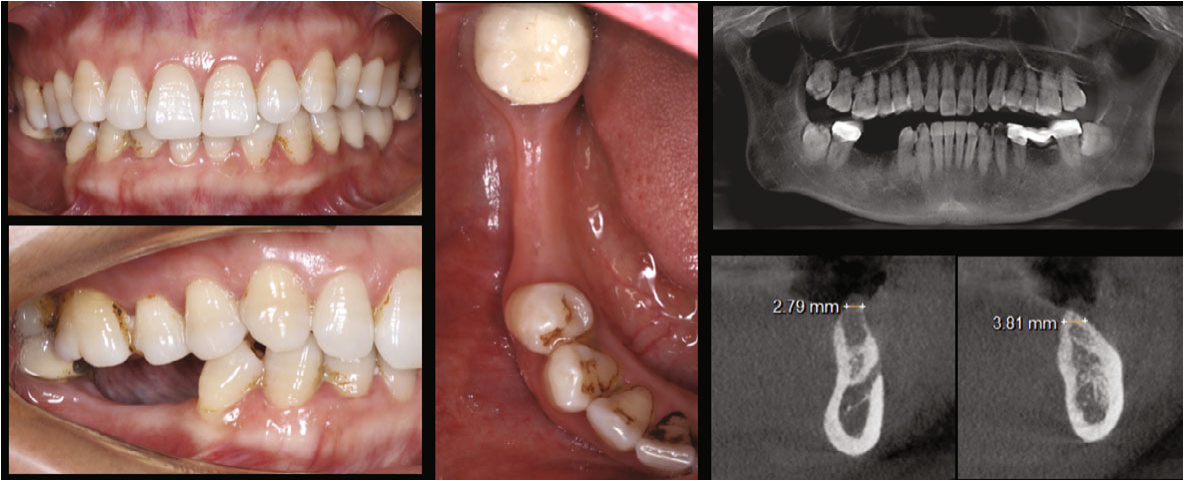
Clinical and radiographic assessment of the patient at baseline. Notice the limited horizontal bone availability for the placement of implants in positions 4.5 and 4.6.

A 46-year-old female patient, classified as ASA Type 1, visited the dental clinic seeking rehabilitation for the missing lower right second premolar (4.5) and lower right first molar (4.6) (Figure 1), both lost due to endodontic failure 5 years before.

The dental team conducted a comprehensive clinical and radiographic examination (Figure 1), revealing poor plaque control and localized Stage 1 Grade B periodontitis. The patient received detailed information about her condition, and tailored periodontal therapy was initiated, following the guidelines outlined in the recommendations by Sanz et al. [13].

Following the conclusion of periodontal steps 1 and 2, a cone beam computed tomography (CBCT) scan was taken, and the feasibility of implant rehabilitation was evaluated.

### 2.2. Guided Bone Regeneration

**Figure 2:**
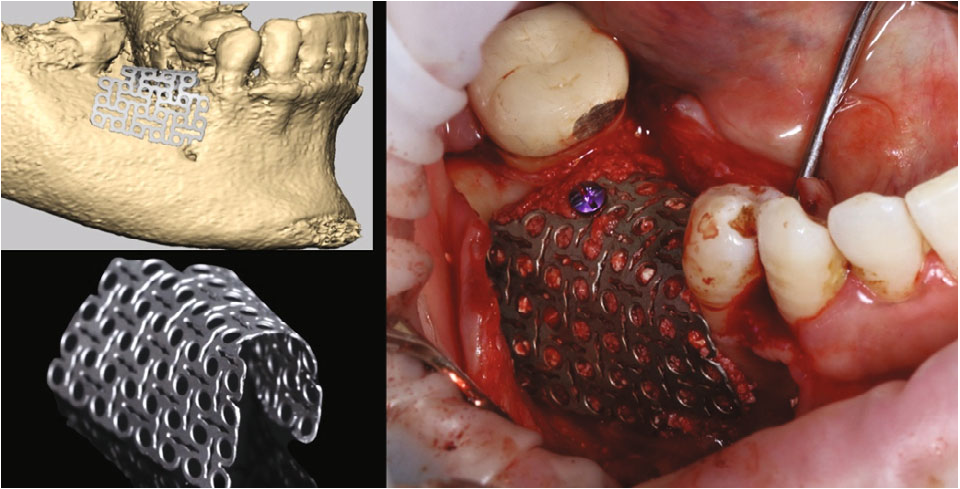
Images of the customized nonresorbable titanium-reinforced membrane design. Clinical image after its fixation in the bone regeneration approach.

In the CBCT analysis, an average bone height of 11 mm from the inferior alveolar nerve and a crest width of 2.79 and 3.81 mm was observed at 4.5 and 4.6 positions, respectively. After assessing the case and determining the need for guided bone regeneration, a personalized approach utilizing a nonresorbable titanium membrane technique was chosen. This membrane, with a thickness of 0.25 mm and a pore size of 1.7 mm, was custom-made based on virtual planning and design using the 3D files obtained from the CBCT scan (Figure 2).

On the day of the surgery, local anesthesia comprising articaine 4% with 1:100,000 epinephrine was administered. An intrasulcular incision was made from the midpoint of tooth 4.3 up to the distal aspect of tooth 4.7, and a crestal incision was done. The position of the titanium mesh was verified, and cortical bone perforations were made to enhance angiogenesis in the regenerated bone [14].

Subsequently, periosteal incisions were performed to mobilize the buccal flap, and the upper fibers of the mylohyoid muscle were detached to facilitate the passivation of the lingual flap and ensure tension-free closure. A mixture of leucocyte- and platelet-rich fibrin (L-PRF) along with xenogeneic bone substitute (Geistlich Bio-Oss, Geistlich Trading Co. Ltd., Beijing, China) was inserted into the mesh and in the bone defect, followed by placement of the mesh into the defect [15]. The mesh was fixed with two 1.6 × 12 mm mini screws (MCT Bio, Gyeonggi-do, South Korea). Once in place, a 30 × 40 mm resorbable collagen membrane (OSTEON 3 Collagen, Dentium China Co. Ltd., Beijing, China) was added to facilitate the attachment of the periosteum to the resorbable mesh.

Wound closure was achieved using horizontal mattress sutures and single interrupted sutures with 5-0 nonresorbable nylon sutures (OEM, Jiangsu, China).

Postoperative instructions were provided, including a prescription for antibiotics and anti-inflammatories (amoxicillin 750 mg every 8 h for 7 days and ibuprofen 600 mg every 8 h as needed). Additionally, it was advised to use 0.12% chlorhexidine mouthwash twice a day for 14 days after the procedure. At this time, sutures were removed.

After 6 months, preoperative digital data was collected, and the CBCT scan of the patient showed a horizontal bone dimension of 8.25 and 9.15 mm in positions 4.5 and 4.6, respectively, and an average vertical dimension of 13 mm.

At that moment, the patient agreed that the autonomous robotic oral surgery system (Remebot, Beijing Rui Yi Bo Technology Co. Ltd., Beijing, China) would be used for the placement of the two dental implants.

### 2.3. Robotic Implant Placement

The CBCT of the patient was imported into robot implant planning software (RemebotDent, Remebot, Beijing Rui Yi Bo Technology DICOM Ltd., China). Thereafter, the placement of two dental implants (CLC CONIC, CLC Scientific, Vicenza, Italy) measuring 4.5 × 10 mm and 5 × 10 mm was planned for positions 4.5 and 4.6, respectively.

Subsequently, a second CBCT scan was performed using a customized resin positioning guide (referred to as the marker). The image plate used for the calibration of the marker showed black-and-white blocks and integrated ambient light detection technology. The marker was strategically positioned in the frontal area of the lower jaw to minimize interference with the operative area, ensuring optimal visibility of the surgical area for the clinician. The information from the positioning marker was transmitted to the robotic surgery system and integrated with the implant planning data.

The crucial 3D areas of interest, like the alveolar ridge and maxillary sinus, underwent segmentation, and meticulous preparation and visualization of the osteotomy plan alongside the target implant position were carried out. Furthermore, an optical tracker was positioned above the patient's head. Ultimately, registration between the robotic arm and the positioning marker was finalized, and calibration was performed.

**Figure 3:**
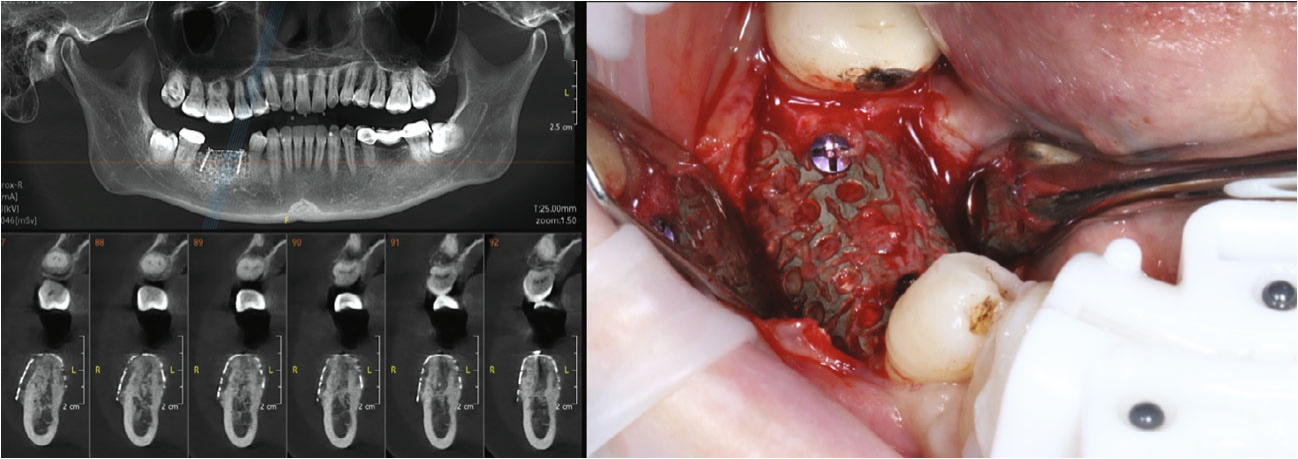
Radiographic and clinical images during the re-entry for implant placement in Positions 4.5 and 4.6. Notice the substantial bone regeneration achieved.

Local anesthesia, consisting of articaine 4% with 1:100,000 epinephrine, was administered and followed by an intrasulcular incision from the midpoint of tooth 4.3 to the distal aspect of Tooth 4.7 to remove the titanium mesh membrane and fixation screws. Clinical confirmation of the significant bone regeneration achieved was also observed (Figure 3).

**Figure 4:**
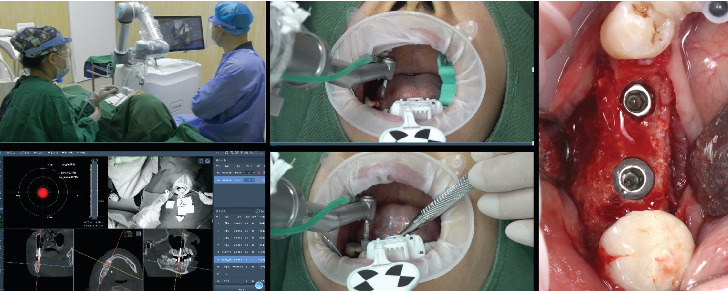
Robot-assisted dental implant surgery. Implants placed in Positions 4.5 and 4.6.

During the operative phase, the surgeon positioned the robotic arm in close proximity to the oral cavity, after which the robotic system automatically adjusted its position to align with the planned implant locations (Figure 4).

Drilling process was executed by the robotic arm following the manufacturer's instructions, and the surgeon withdrew the robotic arm from the patient's mouth. The surgeon could observe the drilling depth, orientation, and force feedback throughout the surgery, enabling real-time monitoring of the drilling position.

Following the preparation of the implant sites, the dental implants (CLC CONIC, CLC Scientific, Vicenza, Italy) were precisely inserted by the robotic system, achieving a final torque exceeding 35 N/cm. Subsequently, the patient underwent a postoperative CBCT evaluation. Once the accurate placement of the implants was confirmed, wound closure was achieved utilizing single interrupted sutures with 5-0 nonresorbable nylon suture (OEM, Jiangsu, China).

Postoperative instructions were provided, including a prescription for antibiotics and anti-inflammatories (amoxicillin 750 mg every 8 h for 7 days and ibuprofen 600 mg every 8 h as needed). Additionally, it was advised to use 0.12% chlorhexidine mouthwash twice a day for 7 days after the procedure. At this time, sutures were removed.

### 2.4. Postoperative Accuracy Analysis

A postoperative CBCT examination was performed to assess the accuracy of implant placement. The DICOM file generated from this examination was imported into the robotic surgery verification system. Within the system, deviations between the planned and actual implant positions were measured, following the methodology outlined in the study by Yang et al. [10]. The verification system provided the following measurements in millimeters: global coronal deviation, vertical coronal deviation, lateral coronal deviation, global apical deviation, vertical apical deviation, and lateral apical deviation. Additionally, the angular deviation was assessed in degrees.

## 3. Results

No adverse events were encountered during the robot-assisted implant surgery, ensuring a smooth and successful procedure. Furthermore, there were no postoperative complications, such as infections or early implant failures, reported. Three months later, assessments of osseointegration revealed promising results. The ISQ values for implants 4.6 and 4.7 were measured at 83 and 78, respectively, indicating optimal osseointegration.

**Figure 5:**
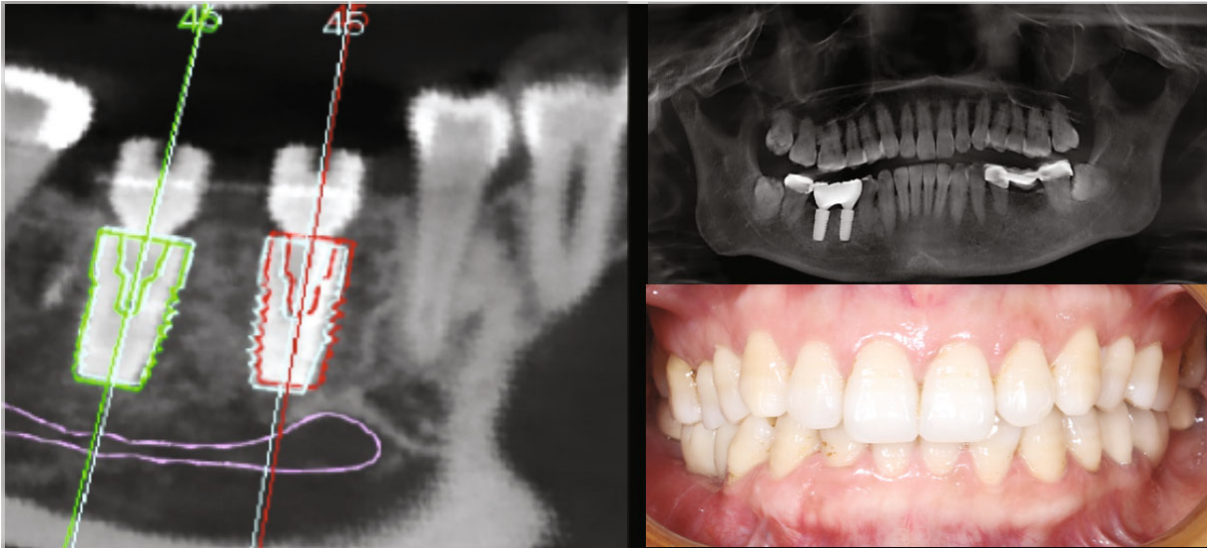
Differences between the intended (planned) and actual (postsurgery) positions of the implants and the final posttreatment radiograph and clinical image of the definitive restoration.

The preoperative virtual and postoperative DICOM files were uploaded to the surgery verification software (Remebot, Beijing). These files were combined to assess the differences between the intended (planned) and actual (postsurgery) positions of the implants (Figure 5). In this regard, the implant in position 4.5 showed a coronal global deviation of 0.57 mm, a coronal lateral deviation of 0.37 mm, and a coronal vertical deviation of 0.44 mm. The global apical deviation was 0.56 mm, the apical lateral deviation was 0.49 mm, and the apical vertical deviation was 0.44 mm. Moreover, the angular deviation was 0.69°. Similarly, the implant in position 4.6 showed a coronal global deviation of 0.41 mm, a coronal lateral deviation of 0.24 mm, and a coronal vertical deviation of 0.34 mm. The global apical deviation was 0.44 mm, the apical lateral deviation was 0.29 mm, and the apical vertical deviation was 0.34 mm. In this implant, the angular deviation was 0.62°.

## 4. Discussion

Recently, r-CAIS is emerging as an encouraging option to traditional computer-assisted implant surgery (CAIS). It provides the potential to significantly improve the accuracy of implant positioning and concurrently mitigate the risk of surgical complications. As aforementioned, both semiautomatic and automatic robotic systems are available in the market. In the semiactive robotic system, the surgeon maintains continuous control over the operational arm during implant osteotomy. This system implies a long learning curve for the clinician and can potentially result in human errors during the surgery [11, 16]. Conversely, in the automatic robotic system, the robot autonomously performs both the implant osteotomy and placement tasks, while the clinician supervises and intervenes when needed [10, 12, 17].

In this case report, the differences between the intended and achieved positions of the implants were examined and closely resemble findings from a recent prospective study, where 31 implants were installed in partially edentulous patients using a semiautomatic robotic system [11]. Specifically, the study revealed coronal deviations of 0.53 ± 0.23 mm and apical deviations of 0.53 ± 0.24 mm. Nevertheless, it is worth mentioning that the mean angular deviation reported in their study (2.81 ± 1.13°) exceeded that of the implants placed in the presented case. The observed differences could be attributed to the use of distinct robotic systems, since in the present investigation an automatic r-CAIS was employed. In fact, in the present article, we observed an angular deviation that closely resembled the results reported in a case series 1.11° (95% CI: 0.78–1.44°), where the same robot was used [17].

However, several factors may impact the final position of the implants. A recent in vitro study, examining the precision of automatic robotic implant site preparation across various implant sizes, revealed that both the diameter and position of the implant significantly affect the robotic system's accuracy [18]. In this sense, greater angular deviation for 5-mm implants compared to 4 and 3.5-mm implants was observed. In addition, after analyzing the robot's performance on the specific tooth position, a notable deviation between the intended and actual implant positions was identified, primarily focused in the molar region [18].

In the present case report, the r-CAIS technology implemented by an automatic robotic device demonstrated reduced deviations in global coronal, global apical, and angular deviations. In this context, in vitro studies have revealed that the robot-assisted implant surgery group showed significantly more decrease in global coronal deviation than the CAIS group [19]. Regarding the clinical outcomes, a meta-analysis showed that the static guide displayed a platform deviation of 1.1 ± 0.35 mm, an apex deviation of 1.63 ± 0.78 mm, and an angular deviation of 5.02 ± 2.03°. In comparison, the dynamic guide showed a platform deviation of 0.96 ± 0.53 mm, an apex deviation of 1.06 ± 0.59 mm, and an angular deviation of 2.41 ± 1.42° [20]. On the other hand, dynamic navigation showed a horizontal deviation at the implant platform of 0.83 ± 0.55 mm, an apex deviation of 0.91 ± 0.56 mm, and an angular deviation of 1 ± 0.48° [20]. Considering all aspects, the promising accuracy and precision of robotically guided implant placement suggest its potential to deliver reliable and consistent clinical outcomes. Notably, the superior performance of r-CAIS technology, particularly in terms of apical and angular deviations, underscores its clinical value [19, 20]. Moreover, newcomer dentists to the field of implant dentistry can benefit from the enhanced precision, efficiency, and control offered by robotic systems to improve their surgical performance.

However, r-CAIS technology still exhibits several inherent errors [9, 21]. First, several sources of error in r-CAIS technology have been highlighted in the literature, including issues with CBCT data calibration, acquisition, registration, and the robotic arm itself [21]. Furthermore, the accuracy of CBCT can be impacted by factors such as scanning thickness, voxel size, image import, and the resolution of the machine, all of which have implications for preoperative planning [9]. Additionally, errors related to the manufacturing, position, and stability of positioning markers can influence registration and calibration accuracy [11]. Lastly, the movement and positioning errors of the robotic arm serve as direct sources of error, with their accuracy and stability significantly affecting implant placement deviations [11]. Although r-CAIS technology holds promise for achieving optimal implant positioning, it does come with certain limitations [22]. Notably, its utilization often proves more time-consuming than static guides, and the technology is associated with higher costs.

The integration of robotics into implantology represents a relatively recent development, with a limited number of publications on automatic and semiautomatic robotic systems currently available [7–12, 17–19, 22]. The accuracy of implant placement using a robotic system is superior to that of freehand surgery and appears to be less influenced by variables such as implant location, jaw type, or implant dimensions [23]. Therefore, further advancements and clinical studies are necessary to enhance the precision, safety, and efficacy of automatic robots in this field.

## Data Availability

The data that support the findings of this study are available on request from the corresponding author. The data are not publicly available due to privacy or ethical restrictions.
